# Olfactory coding in honeybees

**DOI:** 10.1007/s00441-020-03385-5

**Published:** 2021-01-14

**Authors:** Marco Paoli, Giovanni C. Galizia

**Affiliations:** 1 Research Centre on Animal Cognition, Center for Integrative Biology, CNRS, University of Toulouse, 31062 Toulouse, France; 2 Department of Neuroscience, University of Konstanz, 78457 Konstanz, Germany

**Keywords:** Honeybee, Olfaction, Olfactory system, Olfactory coding, Olfactory learning

## Abstract

With less than a million neurons, the western honeybee *Apis mellifera* is capable of complex olfactory behaviors and provides an ideal model for investigating the neurophysiology of the olfactory circuit and the basis of olfactory perception and learning. Here, we review the most fundamental aspects of honeybee’s olfaction: first, we discuss which odorants dominate its environment, and how bees use them to communicate and regulate colony homeostasis; then, we describe the neuroanatomy and the neurophysiology of the olfactory circuit; finally, we explore the cellular and molecular mechanisms leading to olfactory memory formation. The vastity of histological, neurophysiological, and behavioral data collected during the last century, together with new technological advancements, including genetic tools, confirm the honeybee as an attractive research model for understanding olfactory coding and learning.

## Odorants and their meaning

Honeybees strongly rely on olfaction for various aspects of their life, including foraging, mating, navigation, and social interactions. For this, their olfactory system has adapted to detect a myriad of molecules and complex mixtures, creating many different odors. Some odorants are endowed with an intrinsic (innate) value, whereas the majority does not carry meaningful information per se, but can be associated with a food source or with danger, or can be used as olfactory landmark. Honeybees are generalist foragers, i.e., they are not bound to a single flower species to survive. Accordingly, their olfactory circuit is not optimized to detecting the fragrance of a few specific flowers, but to detect, discriminate and learn virtually an infinite number of odorants. However, individual bees forage within a radius of a few kilometers from the hive and for a time span of a few weeks only. As a result, they experience but a limited number of salient olfactory cues, which they must efficiently learn and discriminate.

Wording in olfaction can be confusing. In this review, we use “chemical” or “substance” for a (volatile) stimulus consisting of a single molecule type, “odorant,” “fragrance,” or “scent” for a volatile that can be smelled by the animal, mixture or not, and “odor” for the percept of the animal. Thus, “odorant” refers to a physical stimulus (e.g., “1-hexen-3-ol” or “40–60% mixture of A and B”), “odor” to the psychophysical entity created in the brain (e.g., “cut grass” or “that rewarding flower type”). Different odorants may elicit the same odor, or the same odorant may elicit different odor percepts in particular situations. As an analogy: in color vision, the physical stimulus would be described as a spectrum across wavelengths, while the percept would be the color (“blue” or “yellow”), and here too, the same spectrum might create different colors depending on the circumstances.

The most common chemicals in floral scents are fatty acid derivatives (e.g., alcohols, aldehydes, ketones), benzenoids (e.g., methyl-2-hydroxybenzoate, benzaldehyde, benzyl alcohol), and terpenoids including sesquiterpenes (e.g., limonene, linalool, ocimene) (Knudsen et al. 1993). Some floral odorants can be innately attractive, such as linalool, 2-phenylethanol and lavender (Nouvian et al. 2015), or may possess an aversive valence if they happen to correspond to innately aversive odorants, such as the bee alarm pheromonal compound isoamyl acetate (Boch et al. 1962), but most plant odorants do not possess any innate valence.

### Pheromones

Pheromones are chemical substances secreted by an animal’s exocrine gland and perceived by another individual of the same species, in which they induce a specific response (Karlson and Lüscher 1959). Primer pheromones induce long-term physiological changes, while signal (or releaser) pheromones elicit a temporary behavior (Wilson and Bossert 1963). Honeybees are eusocial insects and use pheromone-based communication in different aspects of colony ecology, from promoting social cohesion and maintaining an equilibrium across the different castes, all the way to regulating reproduction and swarming (Bortolotti and Costa 2014; Free 1987; Jarriault and Mercer 2012; Slessor et al. 2005).

### Brood pheromones

Behavior and physiology of worker bees can be modulated by a mixture of fatty-acid esters secreted by larvae salivary glands, the brood pheromone. Among the main components of this blend we find methyl and ethyl palmitate, oleate, stearate, and linoleate. Non-volatile compounds are distributed within the hive by physical interaction (Le Conte et al. 1990). The relative amounts of the different components vary with larval stage, and can promote different behaviors in the nurse bees—e.g., cell capping, increasing royal jelly production, accepting new queen larvae (Le Conte et al. 2001), or directly inhibit ovarian development (Mohammedi et al. 1998). Bee larvae produce also a highly volatile pheromone, E-β-ocimene, which disperses throughout the colony inhibiting workers’ sexual maturation (He et al. 2016; Maisonnasse et al. 2009), and regulates the numerical equilibrium of nurses versus forager bees (Le Conte et al. 2001; Sagili et al. 2011).

### Workers’ pheromones

Workers use pheromones to communicate and to influence the activity of other workers. When honey receiving bees within the hive cannot get rid of their honey, the ethanol produced by nectar fermentation within their body is transformed in ethyl oleate, a low-volatility pheromone that can diffuse at short range or by physical contact, and provides a foraging inhibition signal (Leoncini et al. 2004). Worker bees possess a strongly developed Nasanov gland, located beneath the intersegmental membrane, and responsible for the production, storage, and release of a pheromonal blend (Pickett et al. 1980). Nasanov pheromone provides an attractive signal used in various contexts, e.g., colony recognition, social cohesion within the hive, or guidance to the entrance of a new nest (Avitabile et al. 1975; Free 1987). Scouts recognize the presence of Nasanov pheromone in a potential nest, which indicates that bees have previously occupied the cavity, making it an attractive choice (Schmidt 2001). Foragers exposed the Nasanov gland for longer durations on more rewarding feeders, suggesting a higher amount of released pheromone in presence of a valuable food source, leading to an increase in the visiting rate from other foragers (Fernández et al. 2003; Free and Williams 1983; Koethe et al. 2020).

A chemical with opposite valence and higher volatility is 2-heptanone, produced in large amount by the mandibular glands of foragers (Shearer and Boch 1965). At exceptionally high concentrations, this odorant triggers a defensive response, but at physiological concentrations, it induces a repulsive behavior: during a foraging flight, a bee marks a visited flower with a small amount of this pheromone, signaling to other foragers to avoid the already depleted flower (Giurfa and Núñez 1992; Stout and Goulson 2001). The effect is short-lived, given the high volatility of 2-heptanone, so that by the time the flower has replenished, the pheromone’s effect has vanished. Together, Nasanov pheromone and 2-heptanone have opposite effects, but exploiting their different volatility both could be deposited on the same flower, creating a temporal sequence of first repellence, then attraction—a hypothesis that remains to be tested.

Pheromones may also communicate danger to the colony and trigger a concerted defense response. Alarm pheromones are released by the Koschevnikov gland located within the sting apparatus, and its main active component, isoamyl acetate, provokes a defensive response and stinging behavior (Boch et al. 1962) by lowering the stinging response threshold, mechanistically elicited by increased brain levels of serotonin and dopamine (Nouvian et al. 2018). This triggers coordinated attacks and stinging behavior (Millor et al. 1999), while decreasing general appetitive learning efficiency (Urlacher et al. 2010). Though this response is genetically pre-determined, it is also modulated by environmental factors. For example, it can be reduced by exposure to floral fragrances with high appetitive value, such as linalool or lavender (Nouvian et al. 2015), suggesting that the bee brain integrates information from innately attractive or aversive stimuli to control which of the many possible behavioral responses to opt for.

One of the most fascinating displays of complex behavior in insects is the honeybee dance, with which bees communicate distance, direction, and quality of a food source (von Frisch 1966). Such behavior creates a multisensory experience: it takes place in the darkness of the hive and relies on sounds (Michelsen et al. 1992), vibrations (Tautz 1996), tactile cues (Rohrseitz and Tautz 1999), taste (Farina and Núñez 1991), and olfaction (Thom et al. 2007). Dancing bees were found to release four characteristic volatiles, which were present in higher amount on waggle dancers than in non-dancing foragers or non-foraging worker bees. Three of these compounds (Z-(9)-tricosene, tricosane, and pentacosane) significantly increase foraging flights, thus suggesting that at least one of the messages of this pheromone is to promote foraging activity (Gilley 2014; Thom et al. 2007).

### Queen’s pheromones

The queen bee provides an important regulatory function within the hive, influencing numerous aspects of colony life, such as social cohesion, workers’ fertility, rearing of new queens, and colony swarming (Kocher et al. 2009). Such regulatory activity is largely mediated by pheromones produced by different glands and in variable amounts throughout the queen’s life. The main source of queen pheromones are the mandibular glands, responsible for the production of queen mandibular pheromone, a mixture of several chemicals, among which the strong sexual attractant 9-oxo-2-decenoic acid (9-ODA) (Butler et al. 1962). Queen mandibular pheromone is released by virgin queens during the nuptial flight to attract the male drones (Free 1987; Gary 1962), while within the hive it is used in combination with other pheromonal compounds (Keeling et al. 2003) to recruit workers for feeding and grooming, the so-called retinue behavior. Retinue pheromones have little volatility and require direct contact or close proximity between the queen and the workers to spread. An increase in honeybee population, or an ageing queen, leads to a reduction in perceived QMP concentration throughout the colony, providing an absence-of-queen signal and promoting the rearing of new queens (Slessor et al. 1988; Slessor et al. 2005). Queen pheromone inhibits ovary development in worker bees, thus maintaining the queen as the only reproductive individual in the colony, and regulates colony homeostasis (Hoover et al. 2003). Indeed, by artificially modulating its level within an experimental hive, it is possible to influence comb building activity, development of foragers, defensive behavior, and aversive learning (Jarriault and Mercer 2012; Slessor et al. 2005).

### Drones’ pheromones

During mating season, honeybee drones from multiple colonies gather in congregation areas waiting for the queen arrival (Koeniger and Koeniger 2004). Such gathering takes place before the arrival of the queen, suggesting that it may not be catalyzed by the queen pheromone (Ruttner and Ruttner 1972). Laboratory preference assays suggested that drone-released pheromones may promote their cohesion in the mating congregation (Brandstaetter et al. 2014; Gary 1962). Analogously, sexually mature virgin queens (but not worker bees of the same age) are attracted to the odor of a group of drones (but not of workers), suggesting the role of a putative drone pheromone in attracting mating queens (Bastin et al. 2017). However, the drone aggregation pheromone has not been identified yet.

## Olfactory coding: the problems

### Odorant discrimination and generalization

Olfactory coding must balance two opposite concepts: discrimination and generalization (Sandoz 2011; Shepard 1987). On one hand, a bee must be capable of discriminating subtle differences between two olfactory objects. On the other hand, small differences in odorant composition might separate fragrances that have the same meaning and therefore should be categorized as the same. Variability in natural odorants means that animals cannot always search for physically the same odorant they associated with, say, a nectar-rich flower. Rather, the molecular composition of an olfactory stimulus may vary, e.g., across flowers of the same plant species, with the time of day, with plant development, or due to a recent landing of another pollinator (Wright and Schiestl 2009; Wright and Smith 2004). Honeybees need to be highly sensitive to small variation in the bouquet in some situations, but in other situations also capable to generalize to avoid discarding good flowers because of minor composition differences.

**Fig. 1:**
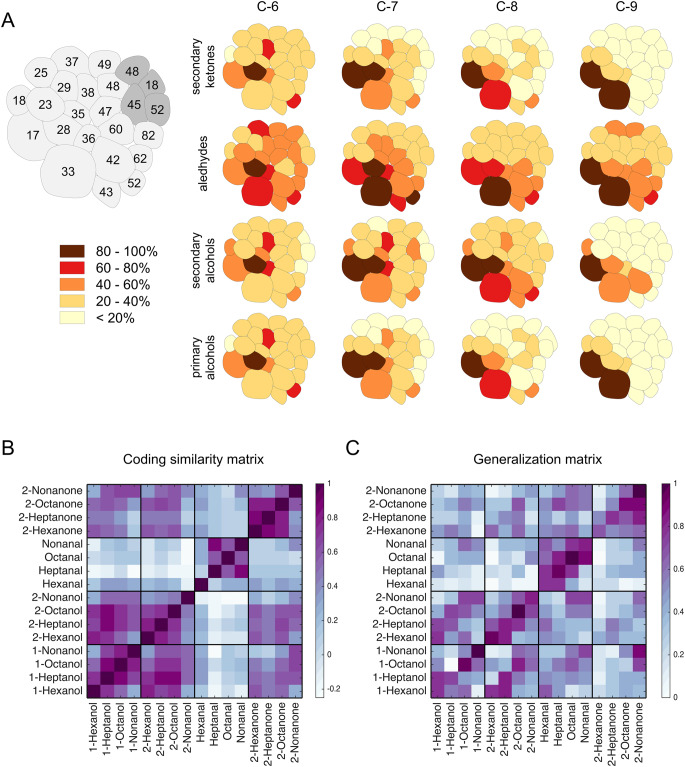
Comparing neurophysiological and perceptual similarity of odorants.** a** Neural correlates of different olfactory stimuli based on Sachse et al. 1999. On the left, a schematic antennal lobe with glomerular labels (light gray, glomeruli innervated by T1 antennal nerve tract; dark grey, glomeruli innervated by T3 antennal nerve tract). On the right, glomerular response maps of 16 odorants, arranged according to their chemical structures (vertical: ketones, aldehydes, secondary and primary alcohols) and carbon chain lengths (horizontal). Response intensity is color coded in five bins, and percentage of glomerular responses is referred to the maximum response intensity to a given odorant. **b** The glomerular response maps in **a** were used to generate an olfactory response similarity matrix based on the Pearson’s correlation coefficient between glomerular response maps. **c** Olfactory generalization matrix based on Guerrieri et al. (2005). After three conditioning trials, 2048 bees were tested for PER to four random stimuli (among which the conditioned one). The response rates to conditioned and novel stimuli are shown on a scale from 0 (= no bee showed PER) to 1 (all tested bees showed PER). A correlation analysis between the two matrices shows that 54% of the behavioral variability can be explained by odorant representation within the antennal lobe (Guerrieri et al. 2005)

What is the generalization function in honeybee olfaction? Smith and Menzel investigated generalization by classically conditioning worker bees to an odorant with multiple rewarded trials, and testing them with a series of novel odorants. They observed that harnessed bees showed high generalization among odorants of the same chemical class, particularly for aldehydes, acetates and monoterpene alcohols (Smith and Menzel 1989). Similarly, using PER conditioning, Guerrieri et al. were able to construct a generalization matrix of 16 odorants varying in chain length and functional groups (Fig. 1), showing that generalization depends on the similarity between stimuli, and it occurs more frequently between long-chained molecules (Guerrieri et al. 2005). In a more naturalistic paradigm, bees were conditioned to an odorant present at a feeder and successively tested in a free-flight choice over 44 vials containing the learnt stimulus and 43 other aliphatic molecules that differed in chain length and functional group (Laska et al. 1999). All bees could correctly identify the trained odorant. Nonetheless, generalization was observed towards odorants belonging to the same chemical group and differing in carbon chain length by only one carbon atom from the conditioned stimulus. Notably, generalization is an asymmetrical phenomenon: a bee may generalize by confusing odorant A with odorant B, but does not necessarily show the opposite generalization behavior from B to A (Guerrieri et al. 2005; Laska et al. 1999).

### Odorant concentration

The same odorant may appear at high concentration (e.g., a bee sitting on the flower collecting nectar), or at low concentration (e.g., when the bee encounters the whiff of that odorant at a distance)—thus, bees should generalize across concentrations. On the other hand, the absolute odorant concentration also contains information, e.g., within a flower about the amount of nectar to be expected. In this situation, a bee should not generalize across concentrations, but she should be able to discriminate among them. Indeed, honeybees can discriminate between different stimulus concentrations—although with faster learning rates at higher concentrations (Wright and Smith 2004)—and are also able to navigate a gradient in search of the learned/rewarded concentration in a non-turbulent environment (Ditzen et al. 2003; Kramer 1976). Notably, while freely moving animals were able to learn absolute concentration levels, restrained animals displayed a higher rate of generalization from high to low concentrations of the same odorant (Bhagavan and Smith 1997; Pelz et al. 1997).

### Odorant mixtures and temporal complexity

Odorants consist of volatile molecules that are transported across space in the air (Mafra-Neto and Cardé 1994; Murlis et al. 1992). These movements are always turbulent: odorant pockets of airborne chemicals form eddies, and at the antenna of the recipient animal (at the olfactory receptors) they create complex temporal sequences of varying concentrations. Bouts of low or zero concentration can alternate with very high or with intermediate concentrations (Celani et al. 2014; Murlis et al. 1992). Due to these temporal complexities, in a turbulent environment, it is not possible to locate an odorant source by gradient ascent. However, the temporal structure of an odorant trail contains some information about the distance from its source, since more distant sources create higher intermittency rates (Riffell et al. 2008). Turbulent distribution of odorant eddies has another important property that animals use to gain information about their environment. Most odor sources release odorant mixtures, and not pure substances. Take, as example, two flowers in a meadow, each with its own characteristic bouquet. In the air, the two mixtures might mix, and thus form a third mixture. How does the bee know that this third mixture is not a third kind of flower? Or that the mixture from a single flower is not a superposition of two other, different sources? The answer lies in the dynamical temporal structure of the olfactory world. Bees exploit the temporal coherence of odorant trails: mixtures that vary consistently in concentration are interpreted as originating from a single source, allowing a bee to separate a target odorant from the olfactory background using the synchronicity of their fluctuation (Hopfield 1991; Nowotny et al. 2013; Szyszka and Stierle 2014; Szyszka et al. 2012). From the neural point of view, the solution might be similar to when humans separate the voice of a person in the noise of a cocktail party (Stierle et al. 2013).

In a natural environment, most olfactory stimuli consist of a bouquet of many substances with unequal relative concentration of the elements. Thus, one of the greatest challenges for understanding olfactory coding is understanding how mixtures are processed: when the components of a mixture blend into a new olfactory object, the perception of the mixture may be considered configural or synthetic (“synthetic coding,” e.g., recognizing a particular Chardonnay and its year); conversely, when a mixture is perceived as the ensemble of its components, we may refer to an elemental (or analytical) processing of the mixture (“elemental coding,” e.g., smelling that there is garlic in a dish) (Kay et al. 2005). By studying honeybee’s ability to discriminate complex mixtures, Laloi et al. showed that not all components are evaluated equally: some of them are more easily identified, suggesting that mixture recognition relies on the identification of a few “key compounds” (Laloi et al. 2000). Similarly, after mixture conditioning, bees were shown to generalize more towards some components than towards others, and that a mixture composed by the key compounds alone could induce the same behavioral response as the whole initial blend, suggesting that a subset of key compounds is sufficient for synthetic coding of complex mixtures (Reinhard et al. 2010). In another study, Locatelli et al. showed that honeybees could distinguish two varieties of snapdragon fragrance. By mimicking natural variations occurring in the two flower blends, they were able to produce novel versions of the two varieties, which bees were able to correctly classify as belonging to one or the other variety. Thus, bees were able to perform a categorization/generalization task including novel odorants in a complex mixture context. Behavioral generalization towards similar blends is supported also by neurophysiological data indicating that similarities among the neural correlates of flowers from the same variety were greater than between different varieties **(**Locatelli et al. 2016**)**. A detailed analysis related the capacity of analytical mixture analysis to the generalization profile of odorants: those substances that induced less generalization were the components that were most dominant within a mixture **(**Schubert et al. 2015**)**.

## Honeybee olfactory system

**Fig. 2:**
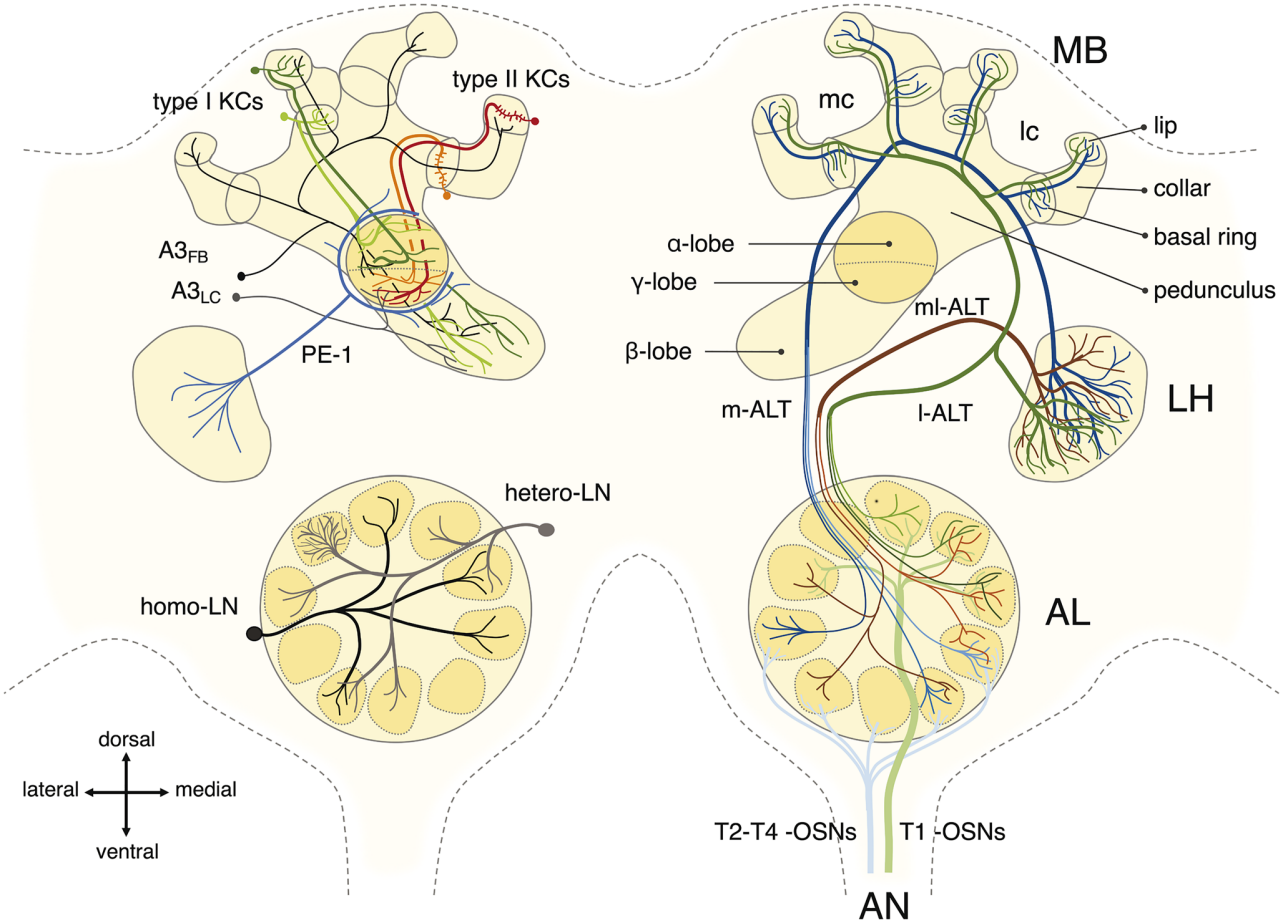
The honeybee olfactory circuit: scheme of the main neuron types of the honeybee olfactory pathway. For clarity, different neuron types and neuronal tracts are labelled with different colors and presented in different hemispheres. Four antennal nerve (AN) tracts (T1 to T4) comprising the axons of ~ 60,000 olfactory sensory neurons (OSNs) innervate the first olfactory neuropil of the bee brain, the antennal lobe (AL). Each OSN innervates a single glomerulus. All OSNs bearing the same olfactory receptor converge onto one of ~ 163 glomeruli, the structural and functional units of the AL. Within the AL, the olfactory input is processed by a local network of ~ 4000 homogeneous and heterogeneous local interneurons (homo- and hetero-LNs), before being relayed to higher order processing centers by the AL output neurons, the projection neurons (PNs). ~ 800 PNs receive uniglomerular input (uniglomerular PNs) and leave the AL via the medial and lateral antennal lobe tracts (m- and l-ALT), two antiparallel tracts projecting to the ipsilateral mushroom body (MB) and lateral horn (LH) of the protocerebrum in opposite order. A second group of projection neurons collects sensory information from multiple glomeruli (multiglomerular PNs), converges into three mediolateral ALTs (here compressed into one for clarity, ml-ALT), and conveys olfactory information to the lateral protocerebrum only. Projection neurons running along the m- and l-ALT innervate the lip and the basal ring of the MB calyces—whereas the collar region is dedicated to the processing of visual input—and synapse onto the MB intrinsic neurons, the Kenyon cells (KCs): ~ 170,000 type I (spiny) KCs innervate the MB calyces with extended and spiny dendritic arborizations, each spine contacting a different PN terminal; ~ 14,000 class II KCs extend short dendrite-like branches ending in clawed specialization forming multiple synapses around a single PN terminal. KCs’ axons descend in parallel fibers from the calyces to the pedunculus: type I KCs bifurcate and innervate the MB ɑ- and β-lobes, whereas type II KCs terminate in the γ-lobe. Inhibitory feedback neurons from the A3 cell cluster (A3_FB_) receive input in the MB lobes and provide a feedback signal to the calyces, acting both on the pre- and post-synaptic site of the PN-KC connections. A second population of A3 extrinsic neurons interconnects the medial and vertical lobes (lobe interconnecting neurons, A3_LC_). From the MB lobes, mushroom body output neurons (MBONs, e.g., the pedunculus extrinsic neuron 1) relay the processed olfactory information to the LH, thus connecting experience-related olfactory information (from the MB) to innate odor information (in the LH)

The olfactory system is a complex neuronal network organized in multiple highly interconnected neuropils. Each of them is a complex neural circuit itself comprising thousands of neurons with feed-back and feed-forward interactions within and across neuropils. Thus, the neural correlate of an olfactory stimulus is the result of local processing within olfactory neuropils and global interactions among neuropils. Below, we provide a detailed review of the neuroanatomy and neurophysiology of the principal neuropils (i.e., “local circuits”) of the honeybee olfactory system (Fig. 2).

### Sensory organs and olfactory receptors

The peripheral olfactory organs of the bee are the antennae: all olfactory sensory neurons (OSNs) are located there. Each antenna is composed of three anatomical structures, from proximal to distal: scapus, pedicel, and flagellum. The flagellum comprises ten flagellomeres (eleven in the drone), the eight more distal of which host the olfactory sensilla: porous cuticular structures containing a variable number of olfactory sensory neurons. Sensilla are classified based on morphology. In the honeybee, the most frequent types of sensilla are placodea, trichodea, basiconica and coeloconica (Esslen and Kaissling 1976; Nishino et al. 2009). The majority of OSNs are housed within sensilla placodea (pore plate sensilla), which are oval-shaped thin cuticular porous plates innervated by up to 35 sensory neurons each (Kelber et al. 2006; Schneider and Steinbrecht 1968). Volatile molecules reaching the honeybee antennae enter the sensillar pores and diffuse through the sensillar lymph until they reach the OSNs’ dendritic membrane. The sensillar lymph prevents the OSNs’ dendritic terminals from drying, and contains olfactory binding proteins (OBPs) and odorant degrading enzymes (ODEs) (Chertemps et al. 2015; Forêt and Maleszka 2006; Iovinella et al. 2018; Song et al. 2018; Vogt et al. 1991; Younus et al. 2014). OBPs are likely involved in facilitating the transition of the odorants from a gaseous to a liquid environment and in transporting them towards the olfactory receptors (Gomez-Diaz et al. 2013; Laughlin et al. 2008; Leal 2013), even if recent studies in *Drosophila* showed that deletion of the principal OBP genes does not impair odor transduction, suggesting that many OBP may not play an essential role in odorant-receptor interaction (Xiao et al. 2019). ODEs, instead, may have a role in the degradation of odorants, thus promoting signal termination by limiting the time an odorant is present in the sensillar lymph and preventing saturation of the olfactory receptors (Iovinella et al. 2018; Leal 2013; Vogt et al. 1985; Younus et al. 2014). Studies in *Drosophila* suggest that ODEs need not be odorant specific, but may be broad spectrum enzymes (e.g., esterases, glutatione-S-transferases, aldehyde dehydrogenases and oxidases, and cytochrome P450s) (Leal 2013; Younus et al. 2017). The neural response spectrum to olfactory stimuli derives both from OR tuning and sensitivity, and from the interaction dynamics with OBPs and ODEs (Larter et al. 2016).

After crossing the hemolymph, odorants reach the OSNs’ dendritic membrane, where they interact with the chemosensory receptors. In bees, most receptors belong to two molecular families: the olfactory (or odorant) receptors (ORs) and the ionotropic receptors (IRs). These receptors are the main drivers for the molecular receptive response ranges of the olfactory sensory neurons. The honeybee genome comprises 163 ORs and 21 IRs (Robertson 2019; Robertson and Wanner 2006). Olfactory receptors are C-terminus-out seven-transmembrane-domain proteins equipped with a ligand-binding domain, and are functionally active as heterodimers (or multimers) with the olfactory receptor co-receptor (Orco) (Benton et al. 2006). In bees, AmOr2 (Krieger et al. 2003) is the ortholog of the fly co-receptor Orco (DmOR38b) (Robertson and Wanner 2006; Sato et al. 2008). While ORs have evolved within the hexapoda clade from gustatory receptors, IRs are more ancient in evolution. They are also multimeric receptor proteins that evolved from ionotropic glutamate receptors, but their physiological properties have not yet been investigated in bees, and our knowledge is extrapolated from *Drosophila* work (Silbering et al. 2011).

The receptor for CO_2_ is not yet known in the bee. In *Drosophila,* a gustatory receptor multimer consisting of DmGR21a and DmGR63a has evolved to form a phasic CO_2_ receptor (Suh et al. 2004). While bees too can detect carbon dioxide (Lacher 1964; Stange and Diesendorf 1973), they do not use a receptor homologous to the fly CO_2_ receptors (Robertson and Kent 2009; Robertson and Wanner 2006; Suh et al. 2004). Within the hive, bees experience levels of CO_2_ far above atmospheric concentration, which they control by ventilating the hive with fanning behavior (Seeley 1974). Carbon dioxide also influences honeybee fertility (Koywiwattrakul et al. 2005; Mackensen 1947), providing a context- and caste-dependent signal able to induce a behavioral effect, such as fanning, and physiological changes, such as modulation of ovary development. For this, they need to detect absolute levels or CO_2_, rather than rapid changes, a necessity that may have led to the independent evolution of a different CO_2_ receptor.

Olfactory receptors’ expression levels vary during honeybee development and are influenced by experience. For example, ORs for linalool and queen pheromone 9-ODA are downregulated after appetitive classical conditioning for those odorants (Claudianos et al. 2014). Furthermore, expression of ORs involved in floral scents detection—but not the co-receptor AmOr2/Orco—show significant variability throughout the year, suggesting an influence of individual experience (Reinhard and Claudianos 2012; Wanner et al. 2007).

### Ligand-binding properties

Vision and hearing are senses that rely on a few continuous dimensions to decode the most important properties of a stimulus, such as intensity, wavelength, frequency, or spatial origin. Chemical senses, and in particular airborne olfaction, employs multiple descriptors (such as molecular size, shape, charge, chain length, presence of functional groups) to identify an odorant and locate it in a multidimensional olfactory space, but which parameters are exploited by olfactory receptors is unknown, and indeed likely to differ among receptors (Münch and Galizia 2016; Sachse et al. 1999). This complexity is exacerbated when we also consider the intensity of an odorant, the multiple components of a mixture, or temporal fluctuations of volatiles.

Olfactory coding initiates with the biochemical interaction between an odorant and the binding pocket of the olfactory receptor, starting odor signal transduction. Many olfactory receptors are activated by multiple odorants and most odorants can interact with more than one receptor, with the specificity of the odorant-receptor interaction influencing the dynamics of the elicited neuronal activity (Getz and Akers 1993; Getz and Akers 1994). This allows for a limited number of receptors to encode a practically infinite number of olfactory combinations (Galizia et al. 1999b; Malnic et al. 1999). Still, every ligand-receptor pair has its own affinity, with higher affinity allowing OR-odorant interaction at lower concentration. Hence, when an odorant is present at low-concentration, it interacts with fewer receptors, while at high concentration also less specific neurons will be recruited, whereas some of the high-affinity OSNs might be saturated and may even stop responding. For this reason, minute variations in stimulus nature and concentration produce significant changes of odor representation across the sensory neurons’ population. It is the task of the olfactory system in the brain to either use these concentration dependent properties for an efficient detection of stimulus concentration, or to ignore their effect for concentration invariance (Strauch et al. 2012).

Floral bouquets may contain enantiomers, i.e., molecules with the same chemical composition but with chiral bonds. Multiple reports have shown that honeybees are capable of discriminating enantiomers for some substances (Aguiar et al. 2018; Laska and Galizia 2001; Lensky and Blum 1974). Although this may appear as a complex task, it is not surprising when considering that odorant detection relies on a ligand sterically interacting with a receptor’s binding pocket, and mirror-like structures do not necessarily fit despite having the same chemical formula, just like left and right foot cannot fit the same shoe. Some studies suggested that honeybees could discriminate between different isotopologues of the same molecule, i.e., molecules that differ, for some of their atoms, in the number of neutrons only (i.e., having different isotopes) (Gronenberg et al. 2014; Paoli et al. 2016a). The only physical mechanism known that could allow for this discrimination would involve reading the ligand’s vibrational spectrum, thus suggesting a mechanism for primary transduction (Turin 1996). However, proving that vibrations are used is experimentally contested: recent experiments in fruit flies revealed that an apparent different perception of isotopologues was in fact caused by minute traces of contaminants in the experimental samples (Paoli et al. 2017). That study showed that a contamination of 0.0006% (6 ppm) was sufficient for a full response, and thus may cause a distorted representation of receptive response profiles.

What is the time required for an odorant to enter the sensillum, reach an OR, and induce a post-synaptic depolarization capable of inducing a measurable signal in the OSNs? By producing olfactory stimuli with high temporal resolution, Szyszka and colleagues measured the response latency from the odorant reaching the antenna to odor signal transduction in the OSNs to be as little as 2 ms **(**Szyszka et al. 2014**)**. Also, electroantennogram responses to high-frequency stimulation showed that honeybee OSNs could follow a stimulus frequency up to 125 Hz. This phenomenon is odorant and concentration dependent, and the coherence between stimulus frequency and antennal nerve firing frequency improved during the stimulus train duration. This does not necessarily imply that a single OSN can follow such a high-frequency stimulation. Instead, it is likely that single neurons may respond intermittently to different pulses of the train of stimuli, and that only their combined activity can outperform the tracking power of a single neuron (Szyszka et al. 2014).

### Antennal lobe

#### Olfactory sensory neurons

All OSNs’ axons converge onto the first processing center of the olfactory circuit, the antennal lobe (AL), functionally analogous and evolutionarily convergent to the vertebrate olfactory bulb (Fig. 2). The honeybee AL is a spheroid neuropil about 300 μm wide, organized in ~ 163 anatomical and functional units called glomeruli (Esslen and Kaissling 1976; Galizia et al. 1999a; Pareto 1972; Robertson and Wanner 2006). Glomeruli are stereotyped in size, shape, and relative position across individuals of the same caste (Sandoz et al. 2007), allowing to build a reference neuroanatomical atlas of the honeybee AL (Flanagan and Mercer 1989b; Galizia et al. 1999a). In the fruit fly, it has been shown that all OSNs expressing the same OR converge onto the same glomerulus, and that this wiring plan is genetically determined (Vosshall et al. 2000). An odorant interacting with different affinities across different ORs elicits neural activity in a subset of glomeruli, creating stereotypical maps of odorant-induced glomerular responses that are, to a large degree, conserved across animals (Fig. 1a). This allowed to complement the morphological atlas with the functional response properties of glomeruli in the honeybee antennal lobe (Galizia et al. 1999b; Sachse et al. 1999) (see also https://neuro.uni-konstanz.de/honeybeeALatlas), which has been used to investigate the principles of olfactory coding (Deisig et al. 2006; Deisig et al. 2010; Paoli et al. 2016b; Paoli et al. 2018; Sachse and Galizia 2003), the relationship between perceptual and chemical similarity of odorants (Carcaud et al. 2012; Guerrieri et al. 2005), as well as changes in olfactory representation upon learning (Chakroborty et al. 2016; Chen et al. 2015; Hourcade et al. 2009; Peele et al. 2006; Rath et al. 2011).

The approx. 60.000 OSN axons enter the AL via the antennal nerve. This nerve enters the brain in six separate subtracts: T1 to T4 are formed by OSNs’ axons and innervate the AL, whereas T5-T6 terminate in the dorsal lobe and the subesophageal ganglion, conveying mechanosensory and gustatory information from the antenna to the brain, and motor information in the opposite direction. Each antennal tract innervates a different subset of glomeruli: tracts T1 and T3 innervate two large groups of approx. 70 glomeruli in the dorsal and ventral AL, respectively, whereas T2 and T4 tracts innervate two minor subsets of 7 glomeruli each (Flanagan and Mercer 1989b). In the honeybee, each antenna innervates only the ipsilateral side (Pareto 1972), unlike *Drosophila* where many axons innervate both ALs. Axons in T4 arborize extensively throughout each innervated glomerulus, while axons from the other tracts innervate the external glomerular layers only, *i.e.* the glomerular cortex (Brockmann and Brückner 1995; Galizia et al. 1999a). Innervation is antennotopic: afferents originating in the distal flagellomers occupy the external margin of the glomerular cortex, proximal segments innervate the inner cortex (Nishino et al. 2009; Pareto 1972). Immunolabelling analysis highlighted the presence of acetylcholinesterase and acetylcholine receptors in the antennal nerve and within AL glomeruli, suggesting that OSN neurotransmission relies on acetylcholine (Kreissl and Bicker 1989).

#### Local interneurons in the antennal lobe

Within AL glomeruli, sensory afferents form synapses with local interneurons (LNs) and projection neurons (PNs). LNs arborize, as a population, throughout the whole glomerular volume and form synapses with both OSNs and PNs (Flanagan and Mercer 1989a; Fonta et al. 1993). Based on their morphology two groups can be classified: homogeneous LNs present a similar density of arborization among all innervated glomeruli, while heterogeneous LNs display a dense arborization in one particular glomerulus and a few sparse branches within the other units they innervate (Flanagan and Mercer 1989a; Fonta et al. 1993; Galizia and Kimmerle 2004; Sun et al. 1993). Based on electrophysiological recordings there are at least five different populations of LNs (Meyer and Galizia 2012; Meyer et al. 2013), an observation confirmed by different populations of peptides being expressed (Galizia and Kreissl 2012). Electrophysiological recordings coupled to cell labeling have shown that LNs’ activity is odor specific, with different stimuli leading to different degrees of excitation or inhibition. Moreover, heterogeneous LNs have a polarized physiology, i.e., they receive input from the densely innervated glomerulus and deliver an output (often inhibitory) to other glomeruli (Galizia and Kimmerle 2004; Sachse and Galizia 2002; Sachse and Galizia 2003). Chemical manipulation of the AL network during olfactory stimulation revealed that the inhibitory effect of LNs is not spatially concentric, but towards glomeruli in a spatially discontinuous pattern (Girardin et al. 2013). Although this may seem inefficient from the connectivity-length point of view, it must be considered that glomeruli are organized on a single layer around the core of the AL, and due to such radial organization, interglomerular distances as seen from the center of the AL are comparable independently of their proximity on the AL surface (Girardin et al. 2013). Alternatively, or in addition, this patchy solution might reflect the multidimensional nature of olfactory coding (Linster et al. 2005): it might not be possible to arrange glomeruli in a two-dimensional or three-dimensional configuration, where interconnectivity creates a logical pattern such that local neuron connectivity is limited to neighboring glomeruli. Honeybees have ~ 4000 LNs (Galizia and Rössler 2010; Witthöft 1967), a relatively large number as compared to *Drosophila* with ~ 200 LNs (Chou et al. 2010; Seki et al. 2010), despite the fact that the ratio of PN count and glomeruli remains somewhat comparable across species (e.g., 185/50 in *Drosophila*, 800/160 in honeybees). In bees, 750 LNs (less than 25% of all LNs) are GABAergic (Schäfer and Bicker 1986), whereas in the fruit fly and cockroaches, the vast majority of LNs are GABA-immunoreactive (Chou et al. 2010; Distler 1989; Leitch and Laurent 1996). Thirty GABAergic LNs constitute a thin commissure linking the two bilateral ALs (Schäfer and Bicker 1986). LNs were also found to be histamine immunoreactive (~ 35 LNs, (Sachse et al. 2006)) or to express different neuropeptides, e.g., allatostatin or tachykinin (Galizia and Kreissl 2012; Kreissl et al. 2010), showing a high functional diversity within the LN population.

Functional imaging analysis of AL input and output signals highlighted the role of the LN local network in olfactory information processing. Although input and output spatial response maps maintain a strong similarity and are dominated by the same odorant-responsive glomeruli, the transition from a compound input signal largely dominated by OSNs’ activity to the output signal of the sole PN population involves a reduction in overall response intensity and a spatial and temporal sharpening of the induced responses. This effect, probably achieved by the inhibitory activity of LNs, contributes to enhancing the contrast between the glomerular representation of different stimuli, and can be strongly reduced by selectively blocking the local inhibitory network **(**Carcaud et al. 2012**; **Carcaud et al. 2018**; **Deisig et al. 2006**; **Deisig et al. 2010**; **Sachse and Galizia 2002**; **Sachse and Galizia 2003**)**.

#### PNs

Olfactory information processed in glomeruli is relayed to higher order brain centers by approx. 800 projection neurons (PNs) (Galizia 2008; Hammer 1997; Rybak 2012). Most of them are uniglomerular (uPNs), receiving input from individual glomeruli, whereas multiglomerular PNs (mPNs) extend their dendritic arborization across multiple glomeruli. The high convergence of ~ 60,000 OSNs onto ~ 800 PNs increases sensitivity and improves signal-to-noise ratio (Galizia 2014). Morphologically, PNs’ dendrites occupy principally the glomerular inner volume, i.e., the core, thus only partially overlapping with the OSNs pre-synaptic terminals which occupy the glomerular cortex (see above) (Galizia et al. 1999a; Pareto 1972). PN axons innervate the mushroom body (MB) and the lateral protocerebrum (LP), in particular the lateral horn (LH) (Fig. 2). Most insects have multiple axonal tracts connecting the ALs to higher order brain areas (Galizia and Rössler 2010). So does the honeybee: axons leave the AL in five AL tracts (ALTs). The lateral (l-ALT) and medial (m-ALT) tracts comprise the axon fibers of uPNs, while mPNs use three smaller mediolateral tracts (ml-ALTs) (Abel et al. 2001; Kirschner et al. 2006; Mobbs 1982). Both the l-ALT and the m-ALT innervate MB and LP, although in reverse order: the former innervates first the LP and then the MB, while the latter does the opposite. m-ALT PNs are strongly acetylcholinesterase immunoreactive and convey olfactory information from T2, almost all T3 and T4 glomeruli **(**Kirschner et al. 2006**; **Kreissl and Bicker 1989**)**. l-ALT fibers receive input mainly from T1 (and from a few T2 and T3) glomeruli **(**Kirschner et al. 2006**)** within the dorsal half of the AL, and exhibit taurine-like (Schäfer et al. 1988) and occasionally GABA-like immunoreactivity **(**Zwaka et al. 2016**)**. This wiring pattern via two separate parallel nerve bundles (l- and m-ALT, respectively), creates different latencies, and is mediated by different neurotransmitters **(**Krofczik et al. 2009**; **Rössler and Brill 2013**)**. Simultaneous labelling of m-ALT and l-ALTs revealed that both pathways remain spatially segregated in the MB: the medial tract innervates the whole lip of the calyces with densely packed pre-synaptic boutons, whereas the lateral one innervates the central core of the lip region with a sparse innervation pattern **(**Kirschner et al. 2006**; **Zwaka et al. 2016**)**. Similarly, they also segregate in the LP, showing a tract-specific compartmentalization **(**Kirschner et al. 2006**)**. Multiglomerular PNs, instead, are mainly GABAergic and project to the LP only **(**Bicker 1999**; **Fonta et al. 1993**; **Schäfer and Bicker 1986**)**.

Fibers of the two tracts differ in response latency, concentration coding, and odorant specificity, suggesting that they may encode different stimulus properties (Brill et al. 2013; Brill et al. 2015; Carcaud et al. 2012; Carcaud et al. 2018; Krofczik et al. 2009; Yamagata et al. 2009). Whereas m-ALT neurons were reported to be more narrowly tuned, prone to elemental mixture analysis, and not subject to mixture inhibition, l-ALT neurons displayed shorter latencies, responded to lower stimulus concentration, tend to encode mixtures synthetically rather than elementally, and were often subject to mixture inhibition (Krofczik et al. 2009; Rössler and Brill 2013). The two tracts may also have different sensitivity/specificity for different odorants, as shown for the processing of some pheromones. Calcium imaging analysis of either m- or l-ALT innervated glomeruli revealed that most odorants activate glomeruli in both areas, including aggregation and alarm pheromones, while queen and brood pheromones activate mostly l- or m-ALT neurons, respectively (Carcaud et al. 2015; Galizia et al. 2012; Müller et al. 2002). A fundamental aspect of a parallel processing system converging on the same neuropils is timing. Recordings of spontaneous and induced activity in both m-ALT and l-ALT simultaneously revealed that the rate of coincident activity was higher in presence of an odorant rather than during spontaneous firing (Brill et al. 2015). However, coincidence rates did not increase between tracts, but only within tracts, particularly among odorant-responsive fibers of m-ALT neurons, which show lower firing frequency but higher coincidence probability than l-ALT neurons (Rössler and Brill 2013).

Although the majority of olfactory processing occurs ipsilaterally, there is some cross-communications in the bee brain: apart from a LNs commissure interconnecting both ALs (see above), uPNs from the T4 glomeruli were shown to project bilaterally to the MB calyces (Abel et al. 2001), and one mPN was reported to innervate multiple glomeruli in both hemispheres and projecting olfactory information from both ALs to the ipsilateral protocerebral lobe (Rybak 2012).

#### Glomerular activity patterns

An odorant reaching the antennae activates a variable number of ORs, thus activating a subset of AL glomeruli, each at a different intensity. These activity patterns depend on ORs’ molecular response ranges, and on network connectivity within the AL. Each odorant elicits a dynamic across-glomeruli pattern, and this stimulus-elicited response creates a trajectory in the glomerular space that is highly reproducible across honeybees. Here, “glomerular space” is the mathematical construct of a multidimensional space where each OSN type represents a single dimension (Fdez Galán et al. 2004). Patterns are largely determined by genetic instructions, and therefore bilaterally symmetrical (Galizia et al. 1998) and comparable across individuals (Galizia et al. 1999b), allowing for the creation of a functional response atlas (see above). Nonetheless, individual variability due to plasticity within the neural network is prominent (Galizia et al. 1999b; Joerges et al. 1997; Sachse and Galizia 2002).

How is stimulus similarity coded in the olfactory circuit at this stage? By means of calcium imaging analysis of the AL, it is possible to record odorant representation across glomeruli, i.e., to determine the location of an odorant in the bee’s glomerular space. This analysis revealed that no single glomerulus represents a particular chemical functional group, and that chemically related odorants evoke partially overlapping response patterns. Considering odorants with the same functional group, their induced glomerular responses gradually shift with changing chain length, and thus their representation in the olfactory space is more similar for similar carbon chain length (Fig. 1). This provided the first evidence how chemical properties of olfactory stimuli were encoded in the brain, and suggested that similar neurophysiological representation could provide the basis for similar odor perception and generalization (Joerges et al. 1997; Sachse et al. 1999). This observation found further support by a direct comparison of the perceptual and neurophysiological distances among pairs of odorants (Carcaud et al. 2012; Carcaud et al. 2018; Guerrieri et al. 2005). A comparison of the behavioral generalization matrix (Guerrieri et al. 2005) and the matrix of neurophysiological distances (Sachse et al. 1999) shows a high correlation (Fig. 1). This observation is also valid for odorant mixtures: Fernandez et al. mixed two odorants varying their ratio, generating a series of binary mixtures. After conditioning honeybees to one of the mixtures, they tested their discrimination ability. They observed that a change in the ratio of the two components induces a proportional change in perceptual similarity, and found a similar gradual shift in the neural representation of the binary mixtures (Fernandez et al. 2009).

Odorant generalization is asymmetric, meaning that bees may generalize more to odorant B after learning odorant A than vice versa (Guerrieri et al. 2005; Sandoz et al. 2001; Smith and Menzel 1989). From the neurophysiological perspective, asymmetric generalization may arise when the pool of receptors activated by two stimuli—and consequently the respective glomerular response maps—are asymmetrically overlapping. In the extreme case where an odorant A activates a sub-group of the receptors activated by B, the activity pattern elicited by the latter encompasses the neural representation of the former. In this case, in a brain model of additive neural computation, A will generalize to B (as a sub-pattern), while B will not generalize to A (since glomeruli present in B do not belong to A).

A similar phenomenon is observed in odorant concentration coding: low concentration (as a sub-pattern) will generalize to high, while high will not generalize to low (since some glomeruli activated at high concentrations do not respond to lower concentrations). Indeed, in behavioral experiments, generalization from low to high concentration is higher than in the other way (Bhagavan and Smith 1997; Pelz et al. 1997). Physiologically, as a general rule, higher concentration leads to the recruitment of more glomeruli, and to signals of shorter latency, higher intensity, and longer duration, resulting in broader and less specific odor response maps (Akers and Getz 1993; Paoli et al. 2018; Sachse and Galizia 2003; Szyszka et al. 2014). The reason is that affinity of an odorant receptor for a ligand depends on their chemical and physical properties, and the likelihood of activation increases with increasing ligand availability. Simultaneous calcium imaging of AL input and output neurons revealed that the AL local network compensates parts of this effect, by modulating the input signal to improve discriminability across concentrations, and yielding a “sharper” odor representation across projection neurons (Sachse and Galizia 2003).

The temporal onset of glomerular responses also contributes to olfactory discrimination (Martin and Hildebrand 2010; Stopfer et al. 1997; Wehr and Laurent 1996) and may be experience and context dependent (Christensen et al. 2000). Fast multiphoton imaging in the honeybee AL showed that each odorant induces a specific latency map, with the ranking of glomerular activity onset highly conserved across individuals. Such ranking-based code has a prediction accuracy comparable to the response amplitude code, thus suggesting that response latencies may provide a first available information for odorant identification, later refined by other informative cues such as the number/type of total responsive units and extent of synchronously firing neurons (Paoli et al. 2018). Electrophysiological recordings investigating AL oscillatory patterns showed that odorous stimuli induce oscillatory synchronization of the projection neurons around 30 Hz detectable both in the AL and in the MB, and likely due to inhibitory feedback loop activity of the LN network (Stopfer et al. 1997). This phenomenon is consistent with an increase of coincident activity across uPNs during and after stimulation (Brill et al. 2015; Galán et al. 2006), as well as with the decrease of other oscillatory frequency powers in AL and MB (Paoli et al. 2016b; Popov and Szyszka 2020). Interestingly, odorant-induced decrease in low-frequency power in the AL does not concern only responsive glomeruli, but also some non-responsive ones (Paoli et al. 2016b), suggesting that this effect is mediated by elements of the local connectivity rather than from a direct interaction of AL input and output neurons.

#### Mushroom body

The MB comprises two cup-like structures, the medial and lateral calyx, which are the converging sites of multimodal sensory inputs, mostly olfactory, visual and gustatory. Each calyx can be subdivided in lip, the main olfactory input region, collar, which receives mainly visual input, and basal ring, which receives input from both sensory modalities (Gronenberg 2001; Mobbs 1982). Axons in the m-ALT innervate a larger volume with higher arborization and synaptic density in the lips than the l-ALT. While ALTs bulk labeling suggested a segregation of l-ALT terminals to the core of the lips, this was not confirmed by single-cell labelling (Kirschner et al. 2006; Zwaka et al. 2016).

MB structure is defined by the morphology of its approx. 184,000 intrinsic neurons, the Kenyon cells (KCs) (Mobbs 1982; Strausfeld 2002), named after F.C. Kenyon who first described them in 1896. Each KC has its cell body inside or around the calyx cup, extends its dendritic arborization within the calyx, and projects its axon into the pedunculus, where it bifurcates into the vertical (α and γ, forming a fused lobe) and the medial (β) lobes (Strausfeld 2002). Topology within the calyx compartments is maintained into the lobes, creating a consistent band pattern in the lobes reflecting the calyx regions (Mobbs 1982; Strausfeld 2002).

Synaptic transmission from PNs to KCs occurs in microcircuits, the microglomeruli (MG). Each MG comprises a PN pre-synaptic bouton surrounded by multiple KC post-synaptic profiles, GABAergic neuron terminals (Ganeshina and Menzel 2001; Groh and Rössler 2020; Grünewald 1999a), as well as modulatory input from octopaminergic and dopaminergic neurons (Blenau et al. 1999; Hammer 1993; Zwaka et al. 2018). The organization of pre- and post-synaptic terminals confers to the MG a spheroidal modular structure, that has been used to label them and quantify how their number and density in different areas of the MB calyces varies, e.g., with age, environmental factors, or after olfactory conditioning (Cabirol and Haase 2019; Groh et al. 2012; Hourcade et al. 2010; Scholl et al. 2014).

KCs are classified in two groups based on their morphology. The main group of approx. 170,000 class I (or spiny) KCs has densely packed somata located within the cups of the calyces. These KCs possess a spiny dendritic tree, each spine taking part in a different MG, thus receiving input from multiple PNs. A second group is composed by approx. 14,000 class II (or clawed) KCs. Their cell bodies are less packed and located just outside the cups of the calyces, and their dendrites extend multiple short claw-like protrusions, each enwrapping a single PN pre-synaptic bouton (Groh and Rössler 2020; Strausfeld 2002). These neuroanatomical differences suggest different functional roles for the two KC populations. While in class I cells a single KC receives inputs from numerous PN boutons and is likely to require coincident inputs to be activated, class II KCs receive multiple synapses from the same PN terminal suggesting that the input of a single neuron may be sufficient to cross its activation threshold. In addition, class I KCs bifurcate and project their axons to both the vertical and the medial lobe, whereas class II neurons innervate exclusively the anterior part of the vertical lobe, i.e., the γ-lobe (Mobbs 1982; Strausfeld 2002). KCs express receptors for acetylcholine, the major neurotransmitter of m-ALT PNs (Kreissl and Bicker 1989). Differently from the fruit fly, where KCs are cholinergic (Barnstedt et al. 2016), histochemical analysis and gene expression studies did not (yet) detect the presence of the cholinergic machinery in bees. Several peptides have been shown in KCs, which may act as co-transmitters, modulators, or “ordinary” transmitters (Kreissl and Bicker 1989; Suenami et al. 2018).

Different types of MB extrinsic (or output) neurons (MBONs) interconnect the mushroom body with other brain neuropils (i.e., unilateral or bilateral output neurons) or interconnect the lobes between them and with the calyces (i.e., recurrent neurons) (Rybak and Menzel 1993). The most well-characterized is a population of ~ 110 A3 GABAergic recurrent interneurons (Grünewald 1999b; Mobbs 1982; Rybak and Menzel 1993; Zwaka et al. 2018). One part of this population innervates a restricted portion of the medial lobe and the corresponding area of the vertical lobe, according to the band pattern innervation characteristic of KCs pre-synaptic innervation (Zwaka et al. 2018). Thus, class I KCs branching in circumscribed regions in both lobes are interconnected by one (or a few) A3 interneurons. The remaining A3 interneurons (also known as MB feedback neurons) receive inputs in the MB lobes and project their axons to the calyces delivering an internal feedback signal to the KCs. They have a stimulus specific activity and extend their pre-synaptic terminals to narrow sub-regions of the calyces, thus providing a stimulus specific pattern of inhibitory activity within the MB input region (Grünewald 1999a; Grünewald 1999b; Zwaka et al. 2018). This compartmentalized tuning system differs from, e.g., the MB feedback network of the fruit fly or the locust, where a single feedback neuron (the anterior paired lateral neuron) collects input from all KCs and enhances sparseness in KC activity patterns by inhibiting the whole KC population (Lin et al. 2014; Papadopoulou et al. 2011). Approximately 150 mushroom body output neurons (MBONs) relay information from the MB lobes to other areas of the protocerebrum (Rybak and Menzel 1993). The dendrites of these neurons branch in large areas of the MB lobes and in the LP, receiving input from different sensory modalities. One of the largest MBONs is the pedunculus-extrinsic neuron 1 (PE-1), an inhibitory neuron collecting mechanosensory, visual and olfactory information (Rybak and Menzel 1998). Neurophysiological studies showed that appetitive olfactory conditioning reduces its inhibitory activity on its target premotor centers (Haehnel and Menzel 2012; Mauelshagen 1993).

#### Lateral protocerebrum

The lateral protocerebrum (LP) and its lateral horn (LH) collect olfactory input both from AL projection neurons and the MB output neurons (Fig. 2). Direct inputs are conveyed by all AL tracts (Kirschner et al. 2006). uPNs forming the m-ALT and l-ALT provide excitatory input from each glomerulus individually, thus conveying the complete AL glomerular response pattern (Roussel et al. 2014). The LP is also targeted by GABAergic mPNs. Within the AL, these neurons collect information across several glomeruli, thus providing an inhibitory input from a glomerular ensemble that may correspond to specific glomerular patterns. Due to their multiglomerular innervation pattern in the AL, they have access to complex across-glomeruli information, which may reflect innate valence. Learned odor information converges on the same areas of the LP from the MB, via the MBONs. These neurons innervate—among other areas of the LP—the LH, and provide experience-related information about the olfactory input (Galizia 2014; Rybak and Menzel 1993).

Whereas within the AL and the MB it is possible to find a prominent neuroanatomical structure, the absence of an evident organization and of anatomical boundaries makes the LP more difficult to investigate. Calcium imaging of the PN dendritic arborizations in the AL and pre-synaptic terminals in the LP revealed that response intensity and intra-odor distances observed in the AL were conserved also in the LP (Roussel et al. 2014), suggesting that PNs innervate the LP in a stereotyped and genetically determined way.

Given that data from bees are limited, we might look at other insects, well aware of the phylogenetic distance between different insect species. Still, because of a similar olfactory coding logic, the fruit fly may help understanding the role of LP in honeybees, and may allow to design working hypotheses to further define its function. In both species, there are by far fewer neurons in the LP than there are KCs in the MB (Aso et al. 2009; Frechter et al. 2019). In flies, the innervation of LP neurons by PNs is not random: excitatory uPNs from the same glomerulus and from similarly tuned glomeruli tend to converge on the same LP neurons, while inhibitory input from broadly tuned mPNs contributes to enhance innate discrimination among similar odorants. PNs innervate the LP in a spatially organized way (Jefferis et al. 2007; Sachse and Beshel 2016; Strutz et al. 2014; Wong et al. 2002). Therefore, the wiring scheme from AL to LP is odorant-biased and has a strong component of genetic predetermination, providing the neural substrate for odorant classification according to their innate valence (Parnas et al. 2013; Strutz et al. 2014). An analysis of odorant coding using principal component analysis revealed that LP neurons are better than PNs in encoding higher-order odor features that are more likely to be behaviorally relevant for the fly (Dolan et al. 2019; Frechter et al. 2019; Jeanne et al. 2018). Hence, it appears that the LP, which does not have the computational power and the architecture to support discrimination and learning of a virtually infinite number of stimuli, may provide a good substrate for innate odorant classification according to behavioral significance. However, also odorants with innate meaning, such as pheromones, are prone to change their valence if experience is strong. For example, bees can be trained to associate the alarm pheromone isoamyl acetate with an appetitive reward (Becker et al. 2000; Sandoz et al. 2001), indicating that worker bees can override their innate aversive reaction. Thus, the innate circuitry within the LP is modulated by the plastic circuitry from the MB (via the connection provided by MBONs), whose input conveys experience-dependent odor valence **(**Galizia 2014**; **Okada et al. 2007**; **Rybak and Menzel 1993**)**.

## Learning and memory

### Olfactory learning

Honeybees have a relatively short foraging life of only about two weeks, while flowers and other food sources are scattered in space and limited in time. They possess a general innate search image characterized by a clear preference for scented and colored feeders (Koethe et al. 2020), but can quickly associate a significant food reward with specific olfactory and visual cues to facilitate future foraging trips (Menzel 1993). Honeybees are polylectic, i.e., they collect nectar and pollen from many different flowering species, but at the same time bees are, to a large degree, flower-constant, i.e., when a flower species is in bloom, they will concentrate on that particular species until depleted, or until a better one comes into blossom. Since a bee cannot know a priori all odorants that it will encounter in her lifetime, learning capacity in the olfactory system is particularly important. Thus, they have evolved a strong learning capability, including an ability to solve a wide range of learning tasks, among which classical and operant learning, context learning, and non-associative learning (Giurfa 2015; Giurfa and Sandoz 2012; Menzel 1993). The possibility to study such behaviors under controlled conditions—both in the laboratory and in the field—allowed investigating the cellular and network-related mechanisms guiding the different phases of memory formation (Eisenhardt 2014; Giurfa 2015; Menzel 1999; Menzel 2012).

Bees learn to associate olfactory cues with information not only in the field, but also within the hive. Karl von Frisch observed that returning foragers were frequently engaging in trophallaxis with other foragers, and by this chemical information transfer they could communicate the nature of the target food source (von Frisch 1965). Analysis of frequency and duration of mandibular contacts during and after a bee dance, suggests that during the dance followers acquire gustatory and olfactory information about the target food source (Farina and Wainselboim 2005; Gil and De Marco 2005). Moreover, a direct contact with a fellow bee may act as a reinforcer even in the absence of nectar (Cholé et al. 2019). Foragers which had not visited the foraging site themselves responded to the odorant associated with the foraging site (Grüter et al. 2006), and feeding to a scented food source increased the preference bias towards the associated odorant (Arenas et al. 2008), suggesting that olfactory information about the food source is transferred between dancer and followers (Farina et al. 2005).

### Classical and operant conditioning

An unconditioned stimulus (US) is a stimulus able to trigger an innate response. A (neutral) conditioned stimulus (CS) does not provide a specific valence per se. In classical conditioning, both stimuli are combined in a temporally organized way, and after the association, the CS is not neutral anymore and elicits the conditioned response (Pavlov 1927). In the honeybee, Pavlovian conditioning is most often used with the proboscis extension response (PER) paradigm upon appetitive association (Bitterman et al. 1983; Kuwabara 1957; Matsumoto et al. 2012; Takeda 1961), and a protocol exploiting the sting extension response upon the exposure to an unpleasant US was recently developed (Giurfa et al. 2009; Junca and Sandoz 2015; Roussel et al. 2010; Vergoz et al. 2007). Conditioned and unconditioned stimuli need to be delivered in temporal contiguity, with the US starting a few seconds later than the CS, so that the CS acquires a predictive value for the US (Bitterman et al. 1983; Szyszka et al. 2011). Conditioning is also possible when there is a temporal gap between the CS and the US, a procedure referred to as trace conditioning (Lüdke et al. 2018; Szyszka et al. 2011). Conditioning can be absolute, where the bee is exposed to a reward-associated stimulus only and learns its predictive value, or differential, where the animal experiences two stimuli, one of which is paired with a reward (CS+) (Bitterman et al. 1983; Matsumoto et al. 2012).

Although most experiments on classical conditioning have used harnessed honeybees, freely moving animals can also be conditioned in flying (Giurfa et al. 1999) or walking (Buatois et al. 2017; Kirkerud et al. 2013; Nouvian and Galizia 2019) paradigms. When the animal is free to move, the learning task might involve components of operant conditioning. In a classical conditioning paradigm, the association between CS and US occurs in a “passive” context, whereas in operant conditioning the animal’s movements adopt a goal-directed component, where learning leads to seeking a reward or avoiding a punishment. Hence, associative learning in an ‘operant’ context reflects the ability of the animal to learn from the consequences of the animal’s own behavior (Brembs 2003).

### Phases of memory formation

Traditionally, memory has been classified by how long it lasts. A single conditioning trial leads to the formation of a short- (seconds to minutes, STM) to medium-term memory (up to 1 h, MTM), whereas multiple conditioning trials lead to the formation of a long-term memory (LTM), which can be retrieved the next day (early LTM) or up to 72 h later (late LTM) (Giurfa and Sandoz 2012; Giurfa et al. 2009; Menzel 2012; Schwärzel and Müller 2006). However, this classification based on time windows was found to be unreliable, with temporal phases being shorter or longer depending on many factors, both external and internal to the animal. A more reliable definition of STM and LTM, therefore, uses the molecular mechanisms involved: STM does not depend on protein synthesis but only on electrical activity or protein phosphorylation (and quickly fades over time), whereas LTM involves protein synthesis: mRNA translation is sufficient for MTM and early LTM formation, but late LTM is both transcription and translation-dependent (Wüstenberg et al. 1998). Honeybees can form late LTM even after a single conditioning trial (Pamir et al. 2014; Sandoz et al. 1995; Villar et al. 2020), and acute injection of transcription and translation blockers showed that transcription but not translation-dependent memory was present already at 4 h, whereas at 24-h single trial-induced memory was already transcription and translation-dependent (Villar et al. 2020).

### Neurophysiology of olfactory learning

All three principal olfactory processing centers (AL, MB, and LP) receive first-order (the AL) or second-order (MB and LP) olfactory information. Also, all three neuropils are innervated by the ventral unpaired median neurons of the maxillary neuromere 1, of which VUMmx1 is the best known (Hammer 1993). At least three reasons suggest the implication of this octopaminergic neuron in appetitive memory formation: (1) it responds to a sucrose stimulation with a long burst of activity, which outlasts the stimulus; (2) it extends its dendritic arborization in the subesophageal ganglion, where it receives gustatory input from sucrose receptor cells, and innervates extensively all odor processing centers; (3) artificial VUMmx1 depolarization paired to an olfactory stimulus induces associative learning of the paired odorant **(**Hammer 1993**)**. Immunohistochemical studies on the distribution of octopamine receptors in the bee brain confirmed its presence in the main odor processing centers. While OSNs do not express the OA receptor genes, antennal lobe PNs and LNs do. The presence of OA receptors in KCs is still unclear, whereas immunoreactive fibers have been found in the MB calyces, possibly belonging to extrinsic MB neurons (Kreissl et al. 1994; Sinakevitch et al. 2011). Compared to the AL and MB, a lower expression of OA receptors is found in the LH (Kreissl et al. 1994; Sinakevitch et al. 2011; Sinakevitch et al. 2013).

During conditioning, the appetitive US triggers octopamine (OA) release (Hammer 1993; Hammer and Menzel 1998). Consistently, injecting OA in the AL or MB can replace the US in appetitive association (Hammer and Menzel 1998), whereas silencing the OA receptor AmOA1 in the honeybee AL impairs memory formation (Farooqui et al. 2003). These experiments revealed that neural plasticity in the AL and in the MB promotes associative learning, and that it relies on the pairing of the olfactory input with OA release. Moreover, OA influences the network activity within the AL to a different degree in different glomeruli, suggesting that the distribution of AmOA1 receptors across glomeruli is not stereotyped, but experience-dependent itself (Rein et al. 2013). Experiments also showed that the system is very robust: while cooling of the entire AL or MB prejudices memory formation (Erber et al. 1980), ablating even large parts of the MBs does not significantly impair memory (Malun et al. 2002).

In the MB, glutamate uncaging experiments showed that glutamate release paired with an olfactory stimulation produces LTM comparable to a classical conditioning protocol (Locatelli et al. 2005). Conversely, silencing NMDA glutamate receptors during (or shortly after) olfactory conditioning impairs memory formation, but affects neither odorant discrimination nor memory retrieval (Müssig et al. 2010). Simultaneous depolarization of the dendritic spines and presence of glutamate are necessary for NMDA receptors to activate. Thus, we may consider a working model where NMDA receptors act as coincidence detectors between CS (i.e., KC depolarization) and US, possibly mediated by glutamatergic MB intrinsic neurons. The opening of NMDA receptor channels produces a sustained increase in intracellular calcium level and the consequent activation of calcium-dependent signaling cascades (Eisenhardt 2014; Jarome and Helmstetter 2013).

The sustained increase in intracellular calcium concentration following the activation of glutamate NMDA receptors or OA receptors leads to an increase in cAMP concentration, and to the activation of protein kinase A (PKA) - CREB signaling cascade (Bollen et al. 2014; Eisenhardt et al. 2003; Leboulle and Müller 2004; Matsumoto et al. 2014; Müller 2000). In addition, multiple CS/US learning trials promote a long-lasting activation of a constitutively active protein kinase C (PKC), a possible mediator of medium and long-term memory related plasticity (Grünbaum and Müller 1998) (for a more in depth review of cellular physiology of memory formation see (Eisenhardt 2014; Himmelreich and Grünewald 2012)). Finally, memory formation results in regulation of gene expression via DNA methylation and demethylation (Biergans et al. 2012; Lockett et al. 2010), with modulatory effects on memory-associated genes (Biergans et al. 2015) and LTM formation (Biergans et al. 2016).

### Learning-related plasticity

Several studies found memory-related changes in glomerular response patterns in the AL (Faber et al. 1999; Locatelli et al. 2013; Rath et al. 2011; Sandoz et al. 2003). An initial analysis of the effects of different appetitive olfactory learning protocols on AL uPNs reported no significant change in odorant-related responses before and after training, thus suggesting that these neurons may be more relevant for consistent odor coding than for memory-related physiological (and possibly morphological) changes (Peele et al. 2006). Nonetheless, traces of non-associative memory could be localized to the AL by optical imaging experiments (Locatelli et al. 2013). A detailed analysis of learning-related physiological changes in across-glomeruli activity patterns allowed to elaborate a working model to explain the events behind associative learning in the AL. The model comprises two superimposed and glomerulus-specific learning effects: a non-associative effect in presence of a pre-synaptic activity not followed by a coincident post-synaptic one (as in the case of an inhibitory LN-to-OSN synapse), and an associative one that relies on coincident pre- and post-synaptic activity (as in the case of an excitatory OSN-to-PN synapse) in presence of an appetitive reinforcement (here, mediated by the octopaminergic VUMmx1 neuron) (Rath et al. 2011). The olfactory system is also modulated by developmental plasticity, where exposure to odors during development leads to morphological changes (Andrione et al. 2017; Devaud et al. 2001; Hourcade et al. 2009; Sachse et al. 2007), or influences their odorant sensitivity and discrimination capability (Jernigan et al. 2020).

Octopaminergic and dopaminergic neurons innervate the MB calyces, relaying appetitive and aversive input. Imaging studies showed that MB neural responses to an odorant increase within the first 30 minutes after conditioning (Faber and Menzel 2001). Associative learning promotes plastic changes in the activity of PN axon terminals: in differential conditioning, the intensity of the responses to both the rewarded (CS+) and the unrewarded stimulus (CS−) may increase or decrease after conditioning, but changes affect mainly CS+ responses, increasing the neurophysiological distance between the two stimuli and the bee’s learning performance. Response reduction appeared more frequently than increase, and the longer latency of inhibitory responses compared to the excitatory ones suggests that learning related plasticity in the MB calyces is, at least in part, driven by inhibitory mechanisms on PNs’ terminals (Haenicke et al. 2018). Simultaneous recordings of PNs and KCs during olfactory stimulation indicated that early-responsive KCs drive the activity of GABAergic feedback neurons, sharpening response dynamics and inhibiting KCs’ responses to late components of the PNs' input signal (Szyszka et al. 2005). Repetitive olfactory stimulation induces a decrease in KCs’ responsiveness (Szyszka et al. 2008)—but not PNs’ input intensity (Peele et al. 2006), suggesting that microglomerular circuits in the MB calyx provide the neural substrate for non-associative memory formation. However, pairing the repeated stimulus with a sugar reward evokes longer-lasting neural responses, promotes the recruitment of additional KCs, and, during the test phase, the response to the conditioned odorant returns to its initial intensity, while the response to the unpaired stimulus remains depressed (Szyszka et al. 2008). In a similar conditioning paradigm, MB feedback neurons—which are both target and input of the KCs—show a decrease of responsiveness, with the response to the CS+ decreasing less than the one to the CS− (Haehnel and Menzel 2010). These experiments suggest the existence of a neural network, where responsive KCs activate a population of MB feedback neurons that exert a general inhibitory activity on the KC population itself. However, the coincidence of CS and US inputs on a KC subpopulation may prevent a decrease in response intensity in those cells and in their downstream targets. LTM formation may also influence MB neuroanatomy: honeybees exposed to paired CS/US stimulation showed an increased density of microglomeruli in the lip, the calyx compartment innervated by olfactory projection neurons, but not in the collar, which receives visual input (Hourcade et al. 2010).

MB output/extrinsic neurons collect processed olfactory inputs from the KCs and relay it to other neuropils, including premotor areas (Rybak and Menzel 1993). Electrophysiology of the vertical lobe output neurons revealed that odor tuning and response strength of a large group MBONs changed after olfactory conditioning (Strube-Bloss et al. 2011). Such plastic changes could not be observed during conditioning itself—thus they are unlikely to be related to the acquisition of a short term memory—but were detected 3 h later, hence supporting MTM/LTM formation. Furthermore, the pedunculus extrinsic neuron 1 (PE-1) innervating the medial and lateral protocerebrum was shown to reduce response intensity to the CS+ specifically, while maintaining unchanged the response to the unpaired stimulus and to a control odorant (Mauelshagen 1993; Okada et al. 2007). These learning-related effects are visible five minutes after conditioning—thus providing a neural substrate for STM—and are possibly due to an increased inhibition of recurrent MB neurons. Olfactory information processed by MB neurons is also collected by the bilateral antennal lobe feedback neuron 1 (ALF-1) that innervates in the vertical lobe in correspondence of the output region of spiny KCs from the calyx lip and projects to the AL with arborizations spanning across the entire neuropil. This neuron has a broad odor tuning and provides a feedback signal to the AL, allowing to modulate olfactory coding at the periphery of the olfactory system (Iwama and Shibuya 1998; Kirschner et al. 2006; Rybak and Menzel 1993).

## Outlook

In this review, we focused on three main aspects of honeybee olfactory coding: (1) the role different odorants play in the life of a honeybee; (2) the neuroanatomy and neurophysiology of the olfactory system; (3) the principal neurophysiological mechanisms leading to memory formation and learning related plasticity. Decades of neuroanatomy, neurophysiology and behavioral experiments provided us with a wealth of knowledge about the logic behind olfactory coding, and of the architecture of the global neuronal network of the olfactory system as well as of the local networks guiding olfactory coding within individual neuropils. Still, a lot remains to do. Luckily, research in honeybee olfaction is alive and vivid, supported by the recent development of large-scale flight tracking, higher resolution optical physiology, and the extensive application of computational tools for the analysis of large behavioral, morphological and neurophysiological datasets. In our view, two areas will be of prominent importance in the next few years, and both areas will strongly benefit from a comparative approach between insect species. The first one relates to understanding the pathway linking stimulus evaluation to decision making. Although we know that different neuropils provide the architecture for different cognitive processes (e.g., the modular MB network allows stimulus recognition and memory storage as well as multisensory integration, the LP may provide stimulus valence evaluation) we still do not fully grasp how learned and innate information are integrated to drive behavior. The second question relates to possible olfactory specialization associated to honeybee sociality. We have reviewed many pheromones that bees use to communicate among conspecifics. Did establishing such a specific mean of communication influence the fundamental neural architecture of the bee brain? As reviewed above, the similarities are plenty even with distantly related species such as the fruit fly, but so are the differences, and it is unclear which of them evolved with honeybee sociality. In this respect, a comparative analysis of social bees with solitary ones, as well as with more distantly related Hymenoptera such as wasps and ants is needed.

## Abbreviations

9-ODA: 9-Oxo-2-decenoic acid
A3: Mushroom body extrinsic neuron of the A3 somata cluster
AL: Antennal lobe
ALF-1: Antennal lobe feedback neuron 1
ALT: Antennal lobe tract
AmOA1: *Apis mellifera* octopamine receptor 1
AmOr2: *Apis mellifera* olfactory receptor 2 (the bee ortholog of the fly co-receptor Orco)
cAMP: Cyclic adenosine monophosphate
CS: Conditioned stimulus
CS+: Reinforced stimulus
CS-: Unreinforced stimulus
DA: Dopamine
DNA: Desoxyribonucleic acid
IR: Ionotropic receptor (olfactory)
KC: Kenyon cell
l-ALT: Lateral antennal lobe tract
LH: Lateral horn in the lateral protocerebrum
LN: Local neuron (in the antennal lobe)
LTM: Long-term memory
LP: Lateral protocerebrum
m-ALT: Medial antennal lobe tract
MB: Mushroom body
MBON: Mushroom body output neuron
MG: Microglomerulus (in the mushroom body)
ml-ALT: Mediolateral antennal lobe tract
mPN: Multiglomerular projection neuron
MTM: Medium-term memory
NMDA: *N*-Methyl-D-aspartate
OA: Octopamine
OBP: Odorant-binding protein
ODE: Odorant-degrading enzyme
OR: Olfactory receptor
Orco: Odorant receptor co-receptor
OSN: Olfactory sensory neuron
PE-1: Pedunculus-extrinsic neuron 1
PER: Proboscis extension response
PKA: Protein kinase A
PKC: Protein kinase C
PN: Projection neuron
QMP: Queen mandibular pheromone
STM: Short-term memory
T1-T6: (Antennal nerve) Tract 1 to 6
uPN: Uniglomerular projection neuron
US: Unconditioned stimulus
