# Correction to: Olfactory coding in honeybees

**DOI:** 10.1007/s00441-026-04065-6

**Published:** 2026-05-27

**Authors:** Marco Paoli, Giovanni C. Galizia

**Affiliations:** 1 Centre des Sciences du Gout et de l’Alimentation, Université Bourgogne Europe, Institut Agro, CNRS, INRAE, 21000 Dijon, France; 2 Department of Neuroscience, University of Konstanz, 78457 Konstanz, Germany

**Correction to: Cell and Tissue Research (2021) 383:35–58**

10.1007/s00441-020-03385-5

The authors regret that the version of Figure 1 that appeared in the original published article is incorrect.

The incorrect Figure 1 appears below.
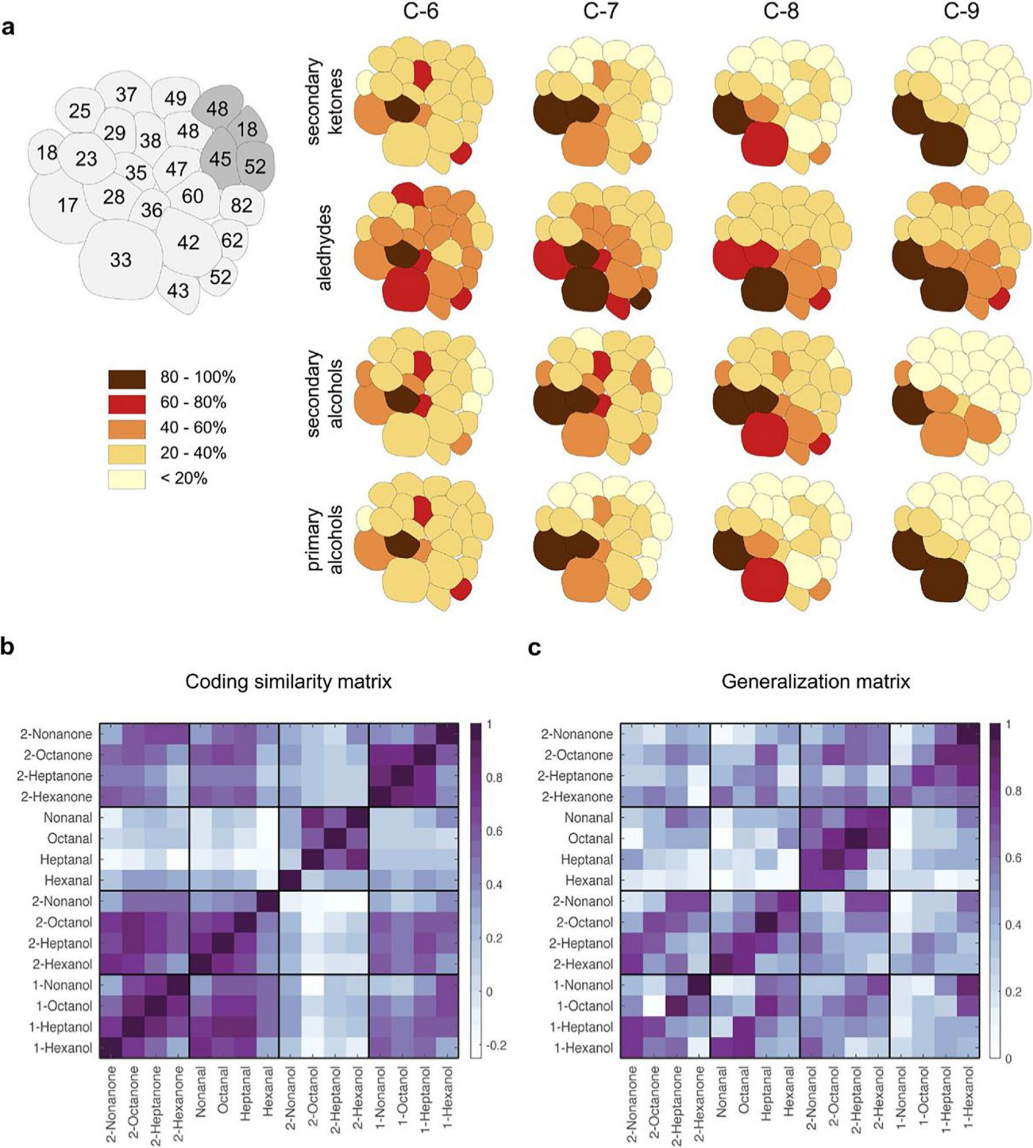


The correct Figure 1 appears below.
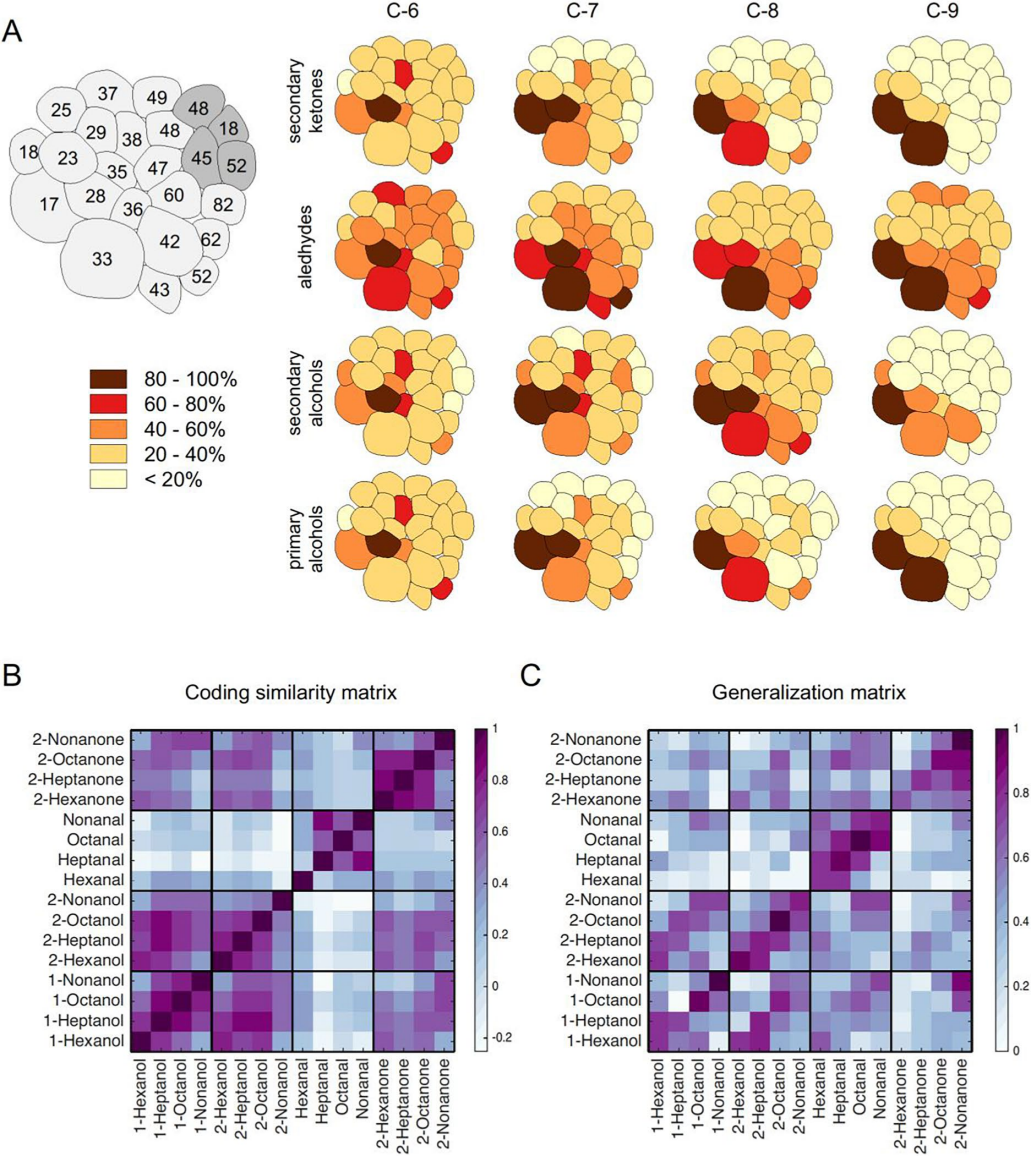


The original article has been corrected.

